# Exploiting the transcriptional specificity of the alpha-methylacyl-CoA racemase *AMACR* promoter for the molecular imaging of prostate cancer

**DOI:** 10.18632/oncotarget.26401

**Published:** 2018-11-30

**Authors:** Mariya Shapovalova, Julia Davydova, Christine Henzler, Mark Daniel, Scott M. Dehm, Christopher A. Warlick, Aaron M. LeBeau

**Affiliations:** ^1^ Department of Pharmacology, University of Minnesota, Minneapolis 55455, MN, USA; ^2^ Department of Surgery, University of Minnesota, Minneapolis 55455, MN, USA; ^3^ Department of Laboratory Medicine and Pathology, University of Minnesota, Minneapolis 55455, MN, USA; ^4^ Department of Microbiology and Immunology, University of Minnesota, Minneapolis 55455, MN, USA; ^5^ Department of Urology, University of Minnesota, Minneapolis 55455, MN, USA

**Keywords:** α-methylacyl-CoA racemase, molecular imaging, adenovirus, prostate cancer

## Abstract

The metabolic protein alpha-methylacyl-CoA racemase (AMACR) is significantly overexpressed in prostate cancer compared to the normal prostate and other non-malignant tissue. Though an attractive target, there are no reports in the literature on leveraging the expression of AMACR for the molecular imaging of prostate cancer. Here, we used a molecular-genetic imaging strategy to exploit the transcriptional specificity of the AMACR promoter for the *in vivo* detection of prostate cancer using the reporter gene luciferase. We performed a stepwise truncation of the promoter and identified a 565 base pair minimal promoter for AMACR that retained both high activity and specificity. Following identification of the minimal promoter for AMACR, we used an advanced two-step transcriptional amplification system to maximize the promoter output. We showed that our optimized AMACR promoter can drive expression of luciferase for molecular imaging in subcutaneous xenograft models of androgen receptor-positive and androgen receptor-negative prostate cancer using a non-replicative adenovirus for gene delivery. Our results provide evidence that the AMACR promoter can be exploited to drive the cancer-specific expression of reporter genes and potentially even be incorporated into conditionally replicative adenoviruses for oncolytic therapy and other applications.

## INTRODUCTION

The isomerase α-methylacyl-CoA racemase (AMACR) is most commonly known for its physiologic role in catalyzing the stereoconversion of the α-methyl proton of branched chained fatty acids undergoing β-oxidation in the mitochondria and peroxisomes [1, 2]. Deficiencies in AMACR protein or activity have been associated with several peroxisomal disorders that lead to neurological impairment due to accumulation of branched-chain fatty acids [3]. The effects of such deficiencies can be ameliorated by decreasing the intake of these lipids that come primarily from meat and dairy-based diets [4]. In the early 2000s, two research groups independently verified AMACR as a prostate cancer (PCa) biomarker based on its specific overexpression in malignant tissue compared to benign prostate tissue by immunohistochemistry (IHC) [5, 6]. Subsequent studies established that AMACR protein was also present in metastatic lesions - not only localized primary PCa - and its expression was independent of the androgen receptor (AR) signaling axis [7–9]. Over the years, AMACR has been established as a dependable biomarker of PCa with IHC analysis finding that AMACR expression in needles biopsies had a 97% sensitivity and 100% specificity for PCa detection [10]. Since its initial discovery in PCa, AMACR overexpression has been documented in a number of other cancers including colon, ovarian and breast [11].

The near-universal overexpression of AMACR in PCa has made it an attractive target for molecular imaging. Due to its overexpression in PCa compared to normal tissue, an AMACR imaging probe can potentially be used to non-invasively differentiate aggressive disease from indolent disease. A number of factors have hindered the development of imaging probes for AMACR. Ideally, an AMACR imaging probe would be a small-molecule inhibitor of its enzymatic activity. There have been a number of studies that tried to develop assays for AMACR detection for high throughput screens of AMACR inhibitors, but none of the inhibitors identified have moved toward clinical application [12–15]. Another complicating factor for a small-molecule imaging probe to be successful is that the probe will have to cross the cell membrane and possibly the membrane of an organelle to reach enzymatically active AMACR. A more favorable approach is a molecular-genetic imaging strategy where the transcriptional specificity of the AMACR promoter is harnessed to drive the expression of reporter genes for cancer detection. The DNA construct containing the promoter and reporter gene can be delivered by viral or non-viral means into the cell where transcription and translation of AMACR are occurring. The reporter genes can encode proteins for a number of imaging modalities including positron emission tomography, magnetic resonance, and bioluminescence imaging [16].

In this study, we detail the development of a molecular-genetic imaging technology for AMACR that can detect PCa *in vivo*. Initially, truncated versions of the full-length 2,295 base pair (bp) AMACR promoter were cloned and analyzed for transcriptional output using a luciferase assay in AR-negative and AR-positive PCa cell lines. From these experiments, we identified a 565 bp minimal AMACR promoter that was cancer-specific and possessed output equal to or greater than the full-length promoter. An advanced two-step transcriptional activation (A.TSTA) system was then used to enhance the output to the minimal AMACR promoter [17]. This system - placed downstream of the minimal AMACR promoter and upstream of luciferase - expresses a GAL4-VP16 fusion protein driven by the minimal promoter. The fusion protein binds GAL4 binding sites upstream of the transcription initiation site that results in an increased transcription of luciferase. Using this system, the output of the minimal promoter was enhanced while still retaining specificity. The enhanced promoter system along with luciferase was then incorporated into a non-replicative adenovirus (Ad) vector. Ad vectors are an efficient natural gene delivery system and are well-researched for cancer gene therapy [18]. The highly efficient delivery of the non-replicative Ad allowed for the imaging of AR-positive and AR-negative PCa xenografts *in vivo* using bioluminescence. Our data provide proof-of-concept that the tissue-specificity of the AMACR promoter can be exploited for detecting PCa via reporter gene imaging. In the future, this strategy could even be applied to therapy by delivering suicide genes or using conditionally replicative adenoviruses for oncolytic and radioviral therapy.

## RESULTS

### AMACR expression in clinical samples and models of prostate cancer

**Figure 1:**
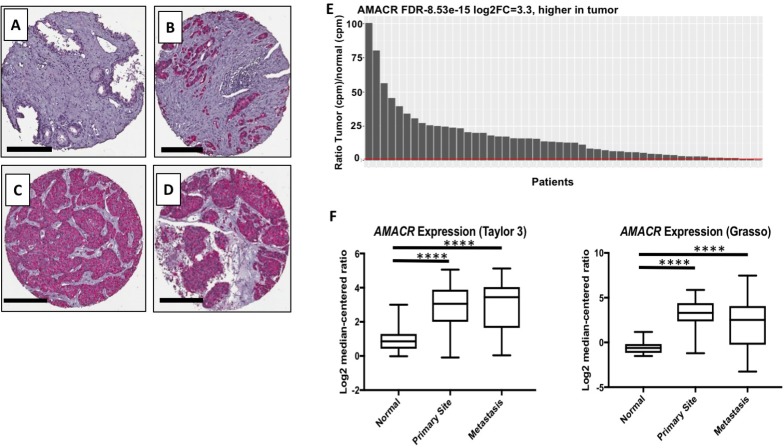
Clinical relevance of AMACR expression in primary and metastatic prostate cancer Immunohistochemical staining of AMACR in healthy prostate (A), prostate adenocarcinoma (B), liver metastasis (C) and adrenal metastasis. Scale bars (**A**–**D**), 200 μm. (**E**) Analysis of the TCGA RNA-seq data documenting that the AMACR expression is overexpressed in the PCa versus normal tissue (*n* = 52). The red bar represents a ratio equal to 1 meaning AMACR expression in both PCa and normal tissue are the same. (**F**) Analysis of the AMACR expression in normal, primary and metastatic PCa from the Taylor and Grasso datasets. Significance was determined using the student *t*-test {^****^p < 0.0001}.

At the protein level, AMACR has been reported in primary and metastatic PCa [5–8]. We confirmed these findings by staining sections from prostatectomy and metastatic lesion biopsies (Figure 1A–1D). As expected, no AMACR was present in healthy prostate tissue (Figure 1A), but intense staining was observed in prostate adenocarcinoma (Figure 1B) and metastatic lesions acquired from liver and lymph node (Figure 1C–1D). It has long been established that concordance between mRNA and protein levels in a cell or tissue is often low (~20%) [19]. Certain proteins are long–lived within the cell requiring infrequent transcription, thus while the protein may be present in the cell, the mRNA may not. For a molecular-genetic imaging strategy to be successful, the tissue-specific promoter must be highly active with high transcriptional rates of the target gene. Though AMACR has been used as a biomarker for IHC for nearly two decades, little analysis of the gene at the transcriptional level has been reported. We analyzed RNA-seq data from three publicly available datasets for AMACR mRNA. In the TCGA [20] dataset comprised of primary PCa samples from 52 patients, we found that AMACR was highly up-regulated in PCa versus normal tissue from the same patient (Figure 1E). Analysis of the Grasso [21] and Taylor [22] datasets found that AMACR was significantly overexpressed in primary and metastatic disease compared to normal tissue, however, no significant difference was observed between primary and metastatic disease (Figure 1F). Analysis of the RNA-seq datasets further supports the cancer-specificity of AMACR and its ubiquitous expression in both primary and metastatic disease. These data also document that significant transcript is present in PCa supporting the use of AMACR transcription machinery for molecular imaging detection of the disease.

**Figure 2:**
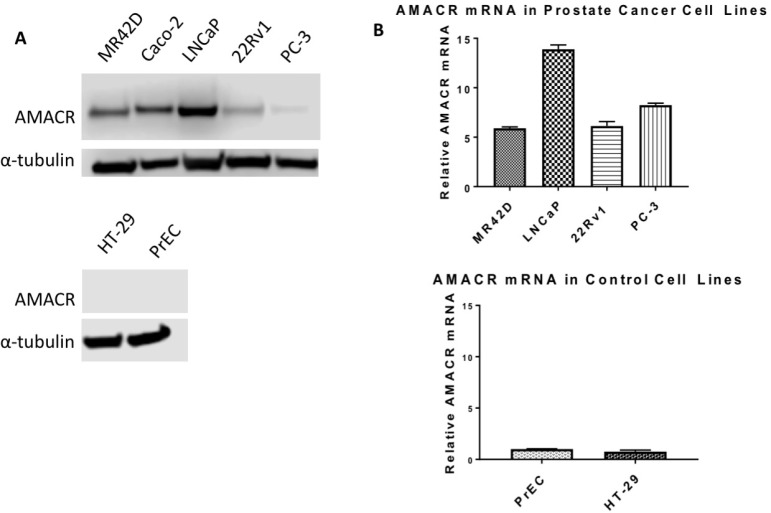
Overexpression of AMACR in prostate cancer cell line models (**A**) Western blot analysis of AMACR protein levels. *Top:* AMACR protein levels in four human PCa cell lines: MR42D, LNCaP, 22Rv1, PC-3, and the positive control colon cancer cell line Caco-2. *Bottom*: AMACR protein levels in prostate epithelial cells (PrEC) and colon cancer HT-29 cells. (**B**) Relative AMACR mRNA levels by qPCR normalized to reference gene 18S ribosomal RNA. *Top*: High AMACR mRNA levels in PCa cell lines. *Bottom*: Low AMACR mRNA levels in healthy PrEC and negative control HT-29 colon cancer cells.

The expression of AMACR at the protein and mRNA levels had previously been reported in LNCaP, PC3 and 22Rv1 cells and we confirmed those expression trends with our results [23–25]. To our knowledge, the expression of AMACR in MR42D cells had not been characterized prior in the literature. LNCaP cells were determined to have the most AMACR protein and mRNA by Western blot analysis and qPCR (Figure 2A and 2B). The AMACR protein band in LNCaP was found to be more intense that of the CaCo-2 cell line, a colon cancer cell line commonly used as a positive control for AMACR (Figure 2A). The level of AMACR protein in the LNCaP-derived castration-resistant MR42D cell line was similar to that of parental LNCaP cells, though mRNA levels differed (Figure 2A and 2B). LNCaP cells are reliant on AR signaling and produce prostate-specific antigen (PSA) whereas the MR42D cell are indifferent to AR signaling, possessing full-length AR, but producing no PSA [26, 27]. We also tested 22Rv1, another castration-resistant model that expresses full-length AR and splice variants, and the highly metastatic AR-negative PC3 cells for AMACR expression [28]. The qPCR results (Figure 2B) indicated that 22Rv1 and PC3 cells may have some inhibition of AMACR protein at the translational level. This was speculated because the AMACR mRNA in 22Rv1 and PC3 was equal or greater than in MR42D, however, by Western blot (Figure 2A), MR42D revealed more protein. As anticipated, no AMACR was detected at either the protein or mRNA level in prostate epithelial cells (PrEC) isolated from healthy prostate tissue and in the colon cancer cell line HT-29 (Figure 2A and 2B). We show that the mRNA levels in 22Rv1, PC3, and MR42D are similar while the protein expression in those cells lines differ. This may be due to translational regulation mechanisms which are outside of the promoter control. These results document that though protein levels may differ potentially, AMACR mRNA is widespread throughout PCa cell lines regardless of AR status.

### Identification and optimization of a minimal promoter for maximum output

**Figure 3:**
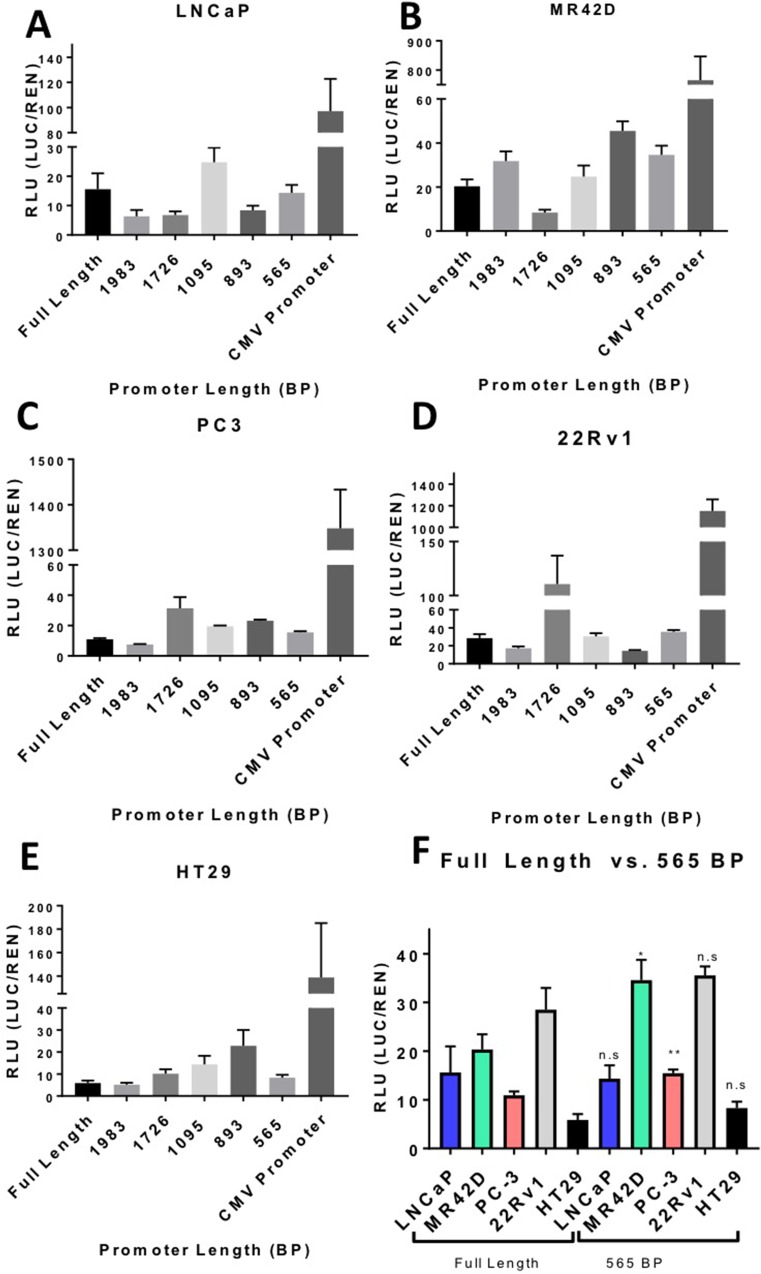
Truncated AMACR promoter analysis by the luciferase assay Transcriptional activity of the truncated AMACR promoters is represented in relative luciferase units (RLU). Luminescence was measure 72 hours post transfection of plasmid containing a promoter and firefly luciferase gene. Luminescence from the firefly luciferase (LUC) driven by the AMACR promoter was normalized for transfection efficiency [co-transfection with pRLTK which expresses renilla luciferase (REN)]. The full-length AMACR promoter and the constitutively-on CMV promoter are presented for comparison for each cell line. (**A**–**D**) AMACR promoter activity in PCa cells. (**E**) AMACR promoter activity in the low AMACR expressing colon cancer HT-29 cells. (**F**) A comparison of the full-length promoter activity in PCa cells and HT-29 cells to the 565 bp truncated promoter. The 565 bp promoter is shown to be equally powerful in LNCap and 22Rv1 cells, more powerful in MR42D and PC-3 cells compared to the full-length promoter and did not show an increase in activity in the low AMACR expressing HT-29 cells. Results are presented as mean ± standard error of the mean (SEM) of *n* = 6. Significance was determined using the student *t*-test {^****^p < 0.0001; ^***^p < 0.001; ^**^p < 0.01; ^*^p < 0.05, n.s. = not significant}.

The full-length 2,295 bp promoter was truncated in a stepwise fashion and the transcriptional efficiency of the truncated AMACR promoters was evaluated using a luciferase assay. The purpose of this assay was to identify a minimal promoter that had a transcriptional output similar to the full-length promoter. Truncations were performed from the 5′ end of the full-length promoter and sites were picked randomly. All of the explored regulatory areas of the promoter based on previous literature search are in the 3′ end and remained untouched. According to Zhang *et al*., 43% of the population has a 20 bp deletion that does not alter the promoter strength [29]. Based on sequencing results, the PBMC donor used for cloning was affected by the 20 bp deletion. Quantitative analysis of the promoter truncations was performed on the PCa cell lines LNCaP, MR42D, PC3, and 22Rv1 were evaluated for transcriptional output with HT-29 serving as a negative control for specificity. The cells were analyzed for luciferase expression 72 hours post-transfection. Several of the truncations, such as 1726 bp and 1893 bp, were found to produce a higher output than the full-length promoter (Figure 3B–3D). The augmented activity of the 1726 bp and 1893 bp promoters was not universal across all cell lines as indicated by the results in LNCaP (Figure 3A). The 565 bp promoter was selected as the minimal promoter for subsequent experiments because it exhibited an output equal to or greater than the full-length promoter and also retained its specificity with little activity in HT-29 cells (Figure 3E, 3F).

**Figure 4:**
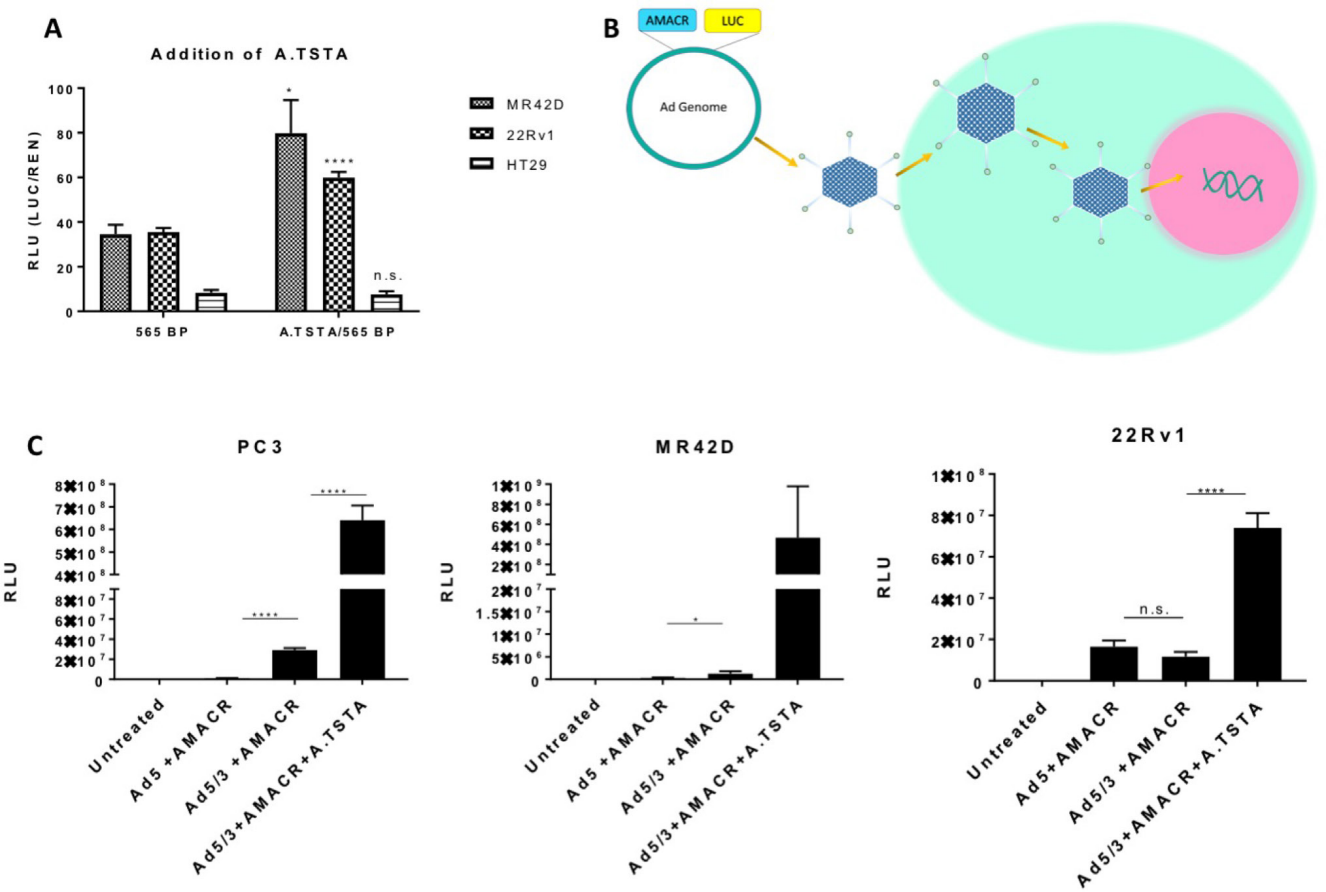
Addition of the advanced two-step transcriptional amplification system and assessment of promoter activity using adenoviral gene delivery *in vitro* (**A**) Luciferase signal 72-hour post transfection of plasmid containing the A.TSTA downstream of the AMACR 565 bp promoter in PCa cells MR42D and 22Rv1, and colon cancer HT-29 cells. The 565 bp promoter data is from Figure 3 and placed here for comparison. Luminescence from the firefly luciferase (LUC) driven by the AMACR promoter was normalized for transfection efficiency [co-transfection with pRLTK which expresses renilla luciferase (REN)]. (**B**) Adenovirus gene delivery to the cells. The promoter or promoter system is introduced to the Ad genome and a virus is constructed. Cells are infected with the virus and DNA is released into the nucleus for gene delivery. (**C**) Promoter activity expressed in RLU (luciferase signal normalized to total protein) 48 hours post-delivery of adenovirus containing firefly luciferase as the reporter gene. The activity of the 565 bp promoter was analyzed using adenovirus serotype 5 (Ad5) and Ad with a chimeric fiber with the knob domain of Ad3 in the Ad5 capsid (Ad5/3) for delivery. The addition of the A.TSTA was analyzed using only Ad5/3 for delivery. RLU is normalized to the protein concentration. Results are presented as mean ± standard error of the mean (SEM) of *n* = 6 in (A) and *n* = 3 in (B). Significance was determined using the student *t*-test {^****^p < 0.0001; ^***^p < 0.001; ^**^p < 0.01; ^*^p < 0.05, n.s. = not significant}.

Tissue-specific promoters such as AMACR often possess relatively weak transcriptional activity, especially when compared to strong viral promoters such as cytomegalovirus (CMV). As a result, this could potentially limit their utility *in vivo*. In order to enhance the transcriptional output of the AMACR minimal promoter without compromising its specificity for PCa, we opted to use a two-step transcriptional amplification (TSTA) system. This system was originally developed by Iyer *et al*. and later was further enhanced by Watanabe *et al*. to create an advanced TSTA (A.TSTA) system [17, 30]. The system is inserted downstream of the promoter and upstream of the gene of interest. An A.TSTA system was used with the AMACR minimal promoter to determine if transcriptional output could be enhanced. The A.TSTA element was inserted in the pGL3 vector containing the AMACR 565 bp promoter and a luciferase assay was performed on 22Rv1, MR42D, and HT-29 cells 72 hours post-transfection. The results documented that the output signal significantly increased in MR42D and 22Rv1 cells compared to the 565 bp promoter data but did not significantly affect HT-29 signal (Figure 4A). The results in (Figures 3, 4A) are on the same scale and (Figure 4A) compares the addition of A.TSTA to the 565 bp promoter data found in (Figure 3). From these data, we can conclude that the addition of the A.TSTA to the minimal 565 bp promoter construct increased transcriptional output without compromising the promoter specificity.

### *In vitro* and *in vivo* adenovirus studies utilizing the AMACR minimal promoter

To further evaluate the strength of the AMACR minimal promoter, we used adenovirus to deliver the reporter construct into cells. Luciferase in the Ad genome was used as the reporter gene to assess the transcriptional efficiency of the promoter (Figure 4B). In this study, only non-replicative Ad was used to assess promoter strength. For comparison, three viruses were constructed: a wild type adenovirus type 5 (Ad5) with the AMACR minimal promoter, an Ad with a chimeric fiber where the tail and shaft domains are Ad5 and the knob domain is of Ad3 (Ad5/3) with the AMACR minimal promoter, and Ad5/3 with the minimal promoter and A.TSTA. PC3, MR42D, and 22Rv1 cells were infected and analyzed for luciferase expression 48 hours post treatment (Figure 4C). We expected to see an increase in signal from cells infected with Ad5/3+AMACR 565 bp compared to the Ad5+AMACR 565 bp based the expanded tropism of Ad5/3. Our findings confirmed that the 5/3Ad was able to enter PC3 and MR42D cells better compared to Ad5, however, no significant difference in luciferase signal was observed when comparing the Ad5 and Ad5/3 in 22Rv1 cells. The addition of the A.TSTA increased the signal significantly in both PC3 and 22Rv1 cells. The infection with Ad5/3+AMACR 565 bp+A.TSTA was shown to not be significant in MR42Ds due to high variability. Based on these results, PC3 and MR42D cells were chosen for *in vivo* xenograft models in the experiment that followed. In summary, (Figure 4C) demonstrated that gene delivery and expression can be improved by modifying the wild type Ad5 fiber to the chimeric Ad5/3 fiber and by adding the A.TSTA system downstream of the AMACR minimal promoter.

**Figure 5:**
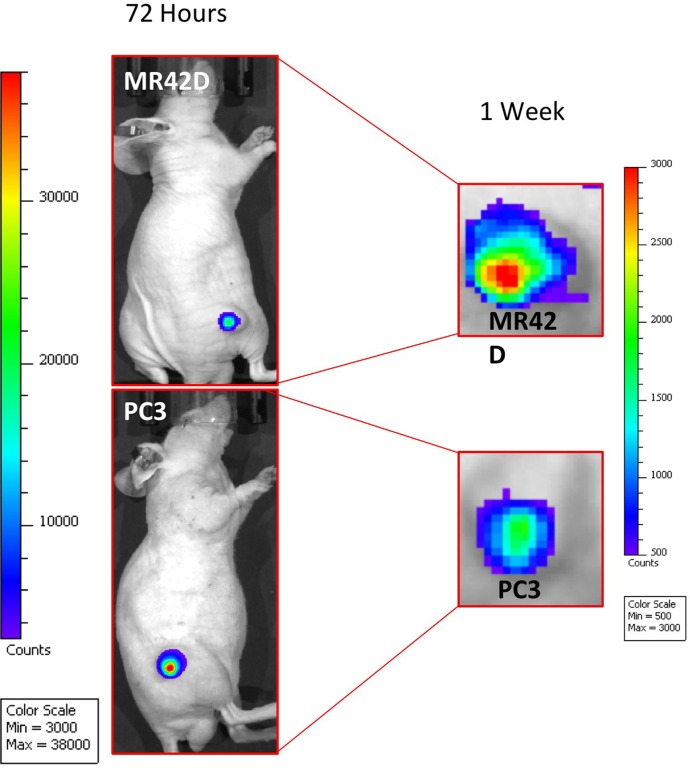
Ad5/3 gene delivery of luciferase guided by the AMACR 565 bp promoter and the A.TSTA system Mice injected intratumorally with Ad5/3+AMACR 565 bp+A.TSTA. The mice were injected with D-luciferin prior to imaging to detect AMACR promoter-driven expression. MR42D and PC3 subcutaneous xenografts in nude mice were used as the PCa models. Images were acquired at 72 hours (left) and at one week (right). The results presented are representative images of *n* = 3 mice. The signal was diminishing by the 1-week time point in both xenografts as demonstrated by the change in the minimum and maximum of the signal strength.

Next, we decided to investigate if the Ad5/3+AMACR 565 bp+A.TSTA could drive the expression of luciferase *in vivo*. MR42D and PC3 cells were used to form subcutaneous tumors in nude mice. Once the tumors reached a volume of 50–100 mm^3^, they were injected with the virus via intratumoral administration and imaged at 72 hours and one week post-injection (Figure 5). Both tumors were bioluminescent at 72 hours. PC3 tumors were observed to be more responsive to the Ad at 72 hours. While the MR42D signal was less intense at 72 hours compared to the PC3, the signal was stronger at the one week time point. This observation suggests that there may be a slower transcriptional onset or less efficient entry to the cells in the MR42D model. The *in vivo* experiment documented that our transcriptional system using the AMACR promoter was powerful enough to have a detectable signal for at least a week after administration of the virus *in vivo*.

## DISCUSSION

The goal of this study was to develop a novel imaging strategy for the detection of PCa using the transcriptional specificity of the AMACR promoter to drive the expression of the reporter gene luciferase. Detecting AMACR can potentially lead to decreased patient overtreatment and the associated co-morbidities and financial costs. Additionally, an AMACR imaging probe delivered intraprostatically could be employed for image-guided biopsy, surgery and focal therapy. Molecular imaging of PCa that exploits the AMACR promoter in this manner has never been investigated prior in the literature. This approach differs from a number of PCa-targeted agents because the promoter, and not the actual protein itself, was used for PCa detection [31]. At the protein level, the expression of AMACR has been identified in both primary and metastatic disease with little to no expression in healthy tissues. Our RNA-seq analysis confirmed the widespread cancer-specific expression of AMACR at the transcript level suggesting that our molecular-genetic imaging strategy can be employed to image both localized and metastatic disease. Another attribute making AMACR an attractive imaging target is that the transcription of the gene is not regulated by the AR. In one investigation, Luo *et al.* found that non-hormone refractory and hormone refractory metastases were strongly positive for AMACR by IHC [8]. Thus, unlike PSA or prostate-specific membrane antigen (PSMA) the level of AMACR expressed will not vary due to androgen deprivation therapy or treatment with second-generation making it a consistent target.

Though not affected by AR modulation, the precise elements that regulate AMACR expression are unknown across PCa cell lines. Previously, an extensive analysis of AMACR promoter activity in different PCa cell lines had not been performed. Chen *et al.* [32]. inspired our promoter truncation experiment with their experiments to determine the promoter regulatory regions. Based on Chen *et al.* and Zhang *et al*. [29]. who determined the CpG island on the 3′ end of the promoter controls gene expression in the colon, we avoided deleting the 3′ regulatory regions. The goal was to determine if there was a shorter, stronger promoter that can behave uniformly in the different PCa cell lines. Our data suggested that the promoter had regulatory sites in the upstream regions that are active in some cells, but activity was variable from cell type to cell type. The minimal 565 bp promoter that we selected for our subsequent studies demonstrated stronger output than the full-length promoter in two PCa the cell lines and the same output as the full length promoter in two other PCa cell lines. Unlike some of the other truncated promoters that had increased signal in the control cells, the 565 bp promoter remained specific for PCa cells. In theory, a shorter promoter would be beneficial for future studies if using plasmid gene delivery as opposed to Ad. Plasmid size is important for delivery because there is a limitation to how much DNA mass can be delivered using polymer transfection reagents. Since delivery using *in vitro* transfection reagent is not as efficient as Ad infection, a shorter promoter would allow more copies of the plasmid delivered. For the purpose of this study, we chose the 565 bp promoter because it had slightly higher transcriptional activity without losing specificity. Enhancing the transcription of the minimal promoter with the A.TSTA system worked especially well when Ad was used as the delivery method.

The efficiency of Ad gene delivery is very dependent on the viral ability to interact with cellular receptors. The wild type Ad5 binds to the coxsackie-Ad receptor (CAR) for entry via its knob domain on the Ad fiber [33]. A strategy in the adenoviral field is the use of chimeric fibers. A well-established method is to use the knob domain of Ad serotype 3, which enters cells independent of the CAR. A vector coding for the Ad3 knob, which binds CD46, was incorporated into the Ad5 genome by Krasnykh *et al*. to create a wild type Ad5 with the original tail and shaft domains of the fiber contain an Ad3 knob [34]. The Ad5/3 with its chimeric fiber expands the tropism of the virus and has been shown to enter cancer cells more efficiently [33]. Our results confirmed the improved targeting of the Ad5/3 in two of the cell lines, PC3 and MR42D. While MR42Ds have never been studied in the context of adenoviral therapy, our PC3 and 22Rv1 findings are consistent with previous literature that found 22Rv1s to be as sensitive to Ad11 as they are to Ad5 and PC3s to be more sensitive to Ad11 than Ad5 [35]. This is relevant because Ad11 binds CD46 for cell entry like Ad3, and therefore the comparison of infectivity between Ad5:Ad5/3 and Ad5:Ad11 should correlate.

While our preliminary results for a very advanced gene therapy approach are promising, there are some limitations to keep in mind. For the purposes of preclinical validation of the AMACR promoter, we used luciferase for PCa detection. If the AMACR promoter is to be pursued further, bioluminescence for PCa detection would have to be replaced with a clinically relevant modality such as positron emission tomography (PET). An example of a reporter gene that can be used for PET imaging is herpes simplex 1 thymidine kinase (HSV1-TK) [36]. HSV1-TK can also be used as a suicide gene for therapeutic purposes [37]. This approach may also have difficulty overcoming tumor heterogeneity and it will not have an effect on detecting necrotic tissue.

In addition to using this molecular-genetic imaging approach for differentiating aggressive PCa from benign disease, our technology can be developed further for therapeutic purposes. In this study, we used a non-replicative Ad to determine whether we can detect the PCa using the AMACR promoter. For therapy, suicide genes such as HSV1-TK or cytosine deaminase could be inserted into the promoter construct in place or adjacent to the reporter gene [37]. Additionally, an oncolytic conditionally replicative adenovirus (CRAd) can benefit from our promoter system to treat PCa. In this scenario, CRAd replication can be guided by the cancer-specific AMACR promoter allowing for tissue-specific replication in the PCa leading to cancer cell death [38]. Our work provides strong evidence that there is value in using the AMACR promoter system in a CRAd and in and also for other Ad based strategies [18] for therapeutic applications.

## MATERIALS AND METHODS

### Immunohistochemistry

Immunohistochemistry was performed on formalin-fixed paraffin-embedded tissue sections using the rabbit anti-AMACR/p504S clone 13H4 antibody (Novus Bio). Unstained sections (4 μm) were de-paraffinized and rehydrated using standard methods. For antigen retrieval, slides were incubated in 6.0 pH buffer (Reveal Decloaking reagent, Biocare Medical) in a steamer for 30 min at 95–98°C, followed by a 20 min cool down period. A serum-free blocking solution (Sniper, Biocare Medical) was placed on sections for 30 min. Blocking solution was removed and slides were incubated in primary antibody diluted in 10% blocking solution/90% TBST. The antibody was used according to the manufacturer's protocol. Patient biopsies for analysis were acquired using a University of Minnesota Human Subjects Division approved IRB protocol for tissue acquisition (IRB#1604M86 269) and with patient consent.

### RNA-seq analysis

The RNA-seq data from the TCGA was analyzed using a method previously described [39]. In short, the data were downloaded from dbGaP, study accession phs000178.v9.v8 [20], yielding paired tumor/normal samples for 52 patients. Genes under 300 bp were removed from further analysis as these are not isolated effectively in standard RNA-seq library preps. Genes with low expression (those with less than 10 reads in half of the samples) were removed, and paired tumor and normal samples were analyzed for differential expression using edgeR.

### Plasmids

For the plasmid promoter luciferase assay, a pGL3 Basic vector (Promega, E1751) was used as the backbone for cloning. For cloning the full-length promoter, peripheral blood mononuclear cell genomic DNA was used. Primers for the different length AMACR promoters can be found in the Supplementary Table 1. The advanced two-step transcriptional amplification system was designed according to Watanabe *et al*. and synthesized by Genscript. An example of a plasmid map used in Figures 3 and 4A can be found in the Supplementary Figure 1.

### Adenoviral vectors

The vectors for adenoviral cloning were provided by the Davydova laboratory. Two adenoviral (Ad) vectors were used, a wild-type pAd5 and the chimeric pAd5/3. Cloning of the Ad vectors was done by homologous recombination with pShuttle vectors containing a firefly luciferase gene and the promoter based transcriptional system. Homologous recombination was performed in BJ5183 electrocompetent cells (Agilent). A total of three viruses were generated for this study (Ad5+AMACR 565 bp, Ad5/3+AMACR 565 bp, and Ad5/3+AMACR 565 bp+A.TSTA). Virus was generated based on Davydova *et al.* [40]. HEK-293T cells were used for viral productions. Cells were transfected with linearized viral vectors (linearized by *PacI*) and delivered to cells using Qiagen Superfect transfection reagent. Cells were observed for the cytopathic effect to determine viral infection. The viruses were amplified and purified by a double cesium chloride density gradient ultracentrifugation and dialyzed in 10% glycerol in PBS. The adenoviral functional titer was determined by using an immunoassay kit from Cell Biolabs.

### Cell lines

The cell lines LNCaP, PC3, 22Rv1, HEK-293T and PrEC were purchased from the American Type Culture Collection (ATCC) and were maintained according to ATCC guidelines. MR42D cells were a gift from Dr. Amina Zoubeidi (Vancouver Prostate Center) and were cultured in 10 mM enzalutamide. HT-29 cells were a gift from Dr. Hiroshi Hiasa (Department of Pharmacology, University of Minnesota). All cell lines were verified by short-tandem repeat analysis and analyzed for mycoplasma contamination prior to our studies.

### Western blot

For protein quantification, 20 μg of protein (lysate) of each cell line were used to run on SDS-PAGE and transferred to a nitrocellulose membrane. The primary polyclonal rabbit AMACR antibody (Sigma, HPA020912) was used at 1:500 and was incubated overnight at 4°C. The blots were analyzed using a LICOR C-DiGit Blot Scanner.

### Quantitative RT-qPCR

10^6^ cells were used for RNA extraction using RNeasy kit (Qiagen). RNA to cDNA conversion was performed using the High capacity RNA to cDNA kit (Applied Biosystems). For gene quantification, Taqman RT-PCR was performed using the Taqman Universal PCR MasterMix (Applied Biosystems) and the following gene expression probes: AMACR Hs01091292 and 18 s ribosomal RNA Hs03928985 for a normalization control. A StepOnePlus Real-Time PCR system instrument (Applied Biosystems) was used for qPCR. Data was analyzed using a comparative Ct method were the fold change = 2 ^−ΔΔCt^).

### Plasmid luciferase assay

10^4^ cells/well were plated in 96 well plates the night before transfecting. Cells were transfected with 90 ng of the experimental plasmid DNA and 9 ng of control pRLTK plasmid (Promega) with 0.24 μl of transfection reagent GeneJuice (Millipore) per well. Cells were analyzed 72 hours post-transfection. For analysis, cells were lysed using the passive lysis buffer from Promega. The Dual-Luciferase Reporter Assay System (Promega) was used to quantify luciferase activity. Each readout of the firefly luciferase (LUC) was normalized to its respective renilla luciferase control readout (REN). Results are reported as LUC/REN = Relative Luciferase Units (RLU).

### Adenoviral luciferase assay

5 × 10^4^ cells/well were plated the day before Ad infection. 0.1 viral particle (vp)/cell in 100 μl media was used. The infection media was replaced after 2 hours with 1mL of fresh media and cells were incubated for 48 hours. At 48 hours cells were lysed using the passive lysis buffer from Promega and the luciferase activity was determined with the Luciferase Assay System (Promega). Luciferase readouts were normalized to the protein content as determined by the Coomassie Plus Protein Assay (ThermoFisher).

### *In vivo* bioluminescence detection

Animal work was done in agreement with our Institutional Animal Care and Use Committee (IACUC) protocol. PC3 and MR42D cells (10^6^) were inoculated into the flanks of nude athymic mice (Envigo) of 3–4 weeks of age in a 1:1 dilution of Matrigel (Corning) to PBS. The tumors were allowed to grow for three weeks. The mice received single intratumoral injections of Ad5/3+AMACR565 BP+A.TSTA (4 × 10^9^ vp in 50 μmL PBS). Images of *in vivo* expression of the luciferase were acquired at 72 hours and 1-week post injection of the virus. For image acquisition, mice were injected intraperitoneally with 150 mg/kg of D-Luciferin potassium salt (GoldBio) and imaged 10 minutes post-injection with the IVIS Spectrum (Caliper/Xenogen). Images were analyzed with Living Image 4.5 software. The min/max values of the signal were not constant for the two imaging timepoints as the signal was significantly lower at the 1-week time point.

## SUPPLEMENTARY MATERIALS FIGURE AND TABLE



## Abbreviations

A.TSTA: advanced two-step transcriptional amplification
Ad: Adenovirus
Ad3: adenovirus type 3
Ad5: adenovirus type 5
Ad11: adenovirus type 11
Ad5/3: adenovirus type 5/3
AMACR: a-methylacyl-CoA racemase
AR: androgen receptor
bp: base pair
CAR: coxsackie-Ad receptor
CMV: cytomegalovirus
CRAd: conditionally replicative adenovirus
IHC: immunohistochemistry
LUC: firefly luciferase
PCa: prostate cancer
PrEC: prostate epithelial cells
PSMA: prostate-specific membrane antigen
PSA: prostate-specific antigen
REN: renilla luciferase
RLU: relative luciferase units
TSTA: two-step transcriptional amplification
