# Correction: Exploiting the transcriptional specificity of the alpha-methylacyl-CoA racemase AMACR promoter for the molecular imaging of prostate cancer

**DOI:** 10.18632/oncotarget.27143

**Published:** 2019-08-06

**Authors:** Mariya Shapovalova, Julia Davydova, Christine Henzler, Mark Daniel, Scott M. Dehm, Christopher A. Warlick, Aaron M. LeBeau

**Affiliations:** ^1^ Department of Pharmacology, University of Minnesota, Minneapolis 55455, MN, USA; ^2^ Department of Surgery, University of Minnesota, Minneapolis 55455, MN, USA; ^3^ Department of Laboratory Medicine and Pathology, University of Minnesota, Minneapolis 55455, MN, USA; ^4^ Department of Microbiology and Immunology, University of Minnesota, Minneapolis 55455, MN, USA; ^5^ Department of Urology, University of Minnesota, Minneapolis 55455, MN, USA

**This article has been corrected:** The proper Grant Support information is shown below:

## GRANT SUPPORT

This research was supported by a Department of Defense Prostate Cancer Research Program Early Investigator Research Award – Predoctoral (MS), Prostate Cancer Foundation Young Investigator Award (AML), Randy Shaver Foundation Research Award (AML), Paul Calabresi NCI K12 Award through Mayo Clinic (AML) and an American Cancer Society Institutional Research Grant (AML).

Original article: Oncotarget. 2018; 9:36693–36704. 36693-36704 . https://doi.org/10.18632/oncotarget.26401

