# Supplementary material for: Exploiting the transcriptional specificity of the alpha-methylacyl-CoA racemase AMACR promoter for the molecular imaging of prostate cancer
Source: Oncotarget. 2018 Nov 30;9(94):36693–704. doi: 10.18632/oncotarget.26401 (PMC6291171; doi:10.18632/oncotarget.26401)
Supplement: Supplementary file 1 [file oncotarget-09-36693-s001.pdf]

Exploiting the transcriptional specificity of the alpha-methylacyl-CoA racemase *AMACR* promoter for the molecular imaging of prostate cancer

SUPPLEMENTARY MATERIALS

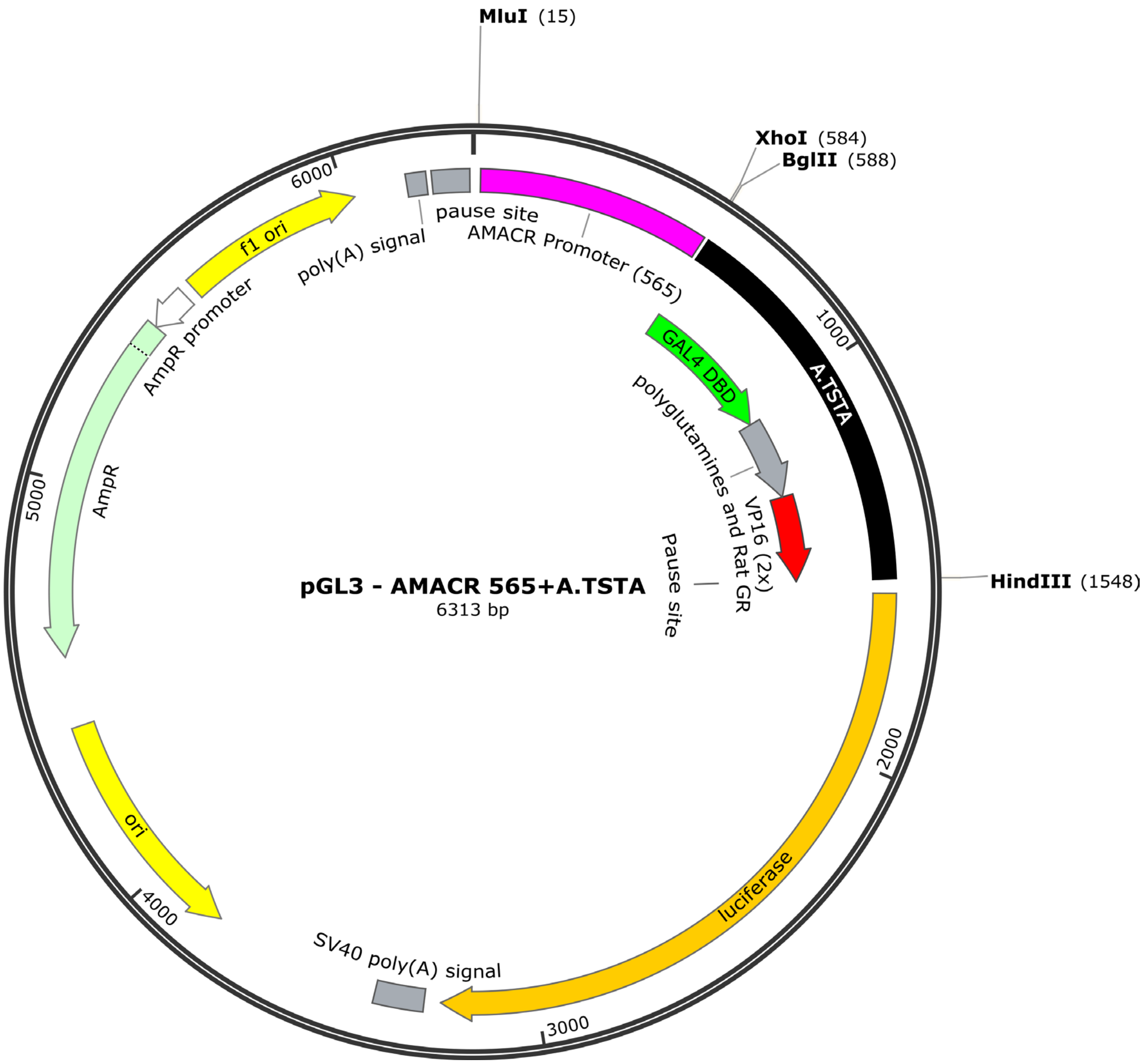

Supplementary Figure 1: Plasmid map. Example of experimental plasmid. The map above shows the plasmid used in Figures 3 and 4A. The promoter was inserted using the MluI and XhoI while the A.TSTA was inserted using BglII and HindIII. The plasmid backbone used was pGL3 from Promega with the firefly luciferase reporter gene.

Supplementary Table 1: Primers for promoter truncation

Primer	Sequence
Reverse (Rev63) – XhoI Cut Site	5′ CGGCTCGAGCTGAAGGAAACTGAGCAG 3′
Full Length (2295 BP) Forward - MluI Cut site	5′ CGACGCGTCCTCAGAAGCATGTGA 3′
1983 Forward – MluI Cut site	5′ GCAACGCGTGGGACTGCTGGATCATAT 3′
1726 Forward - MluI Cut site	5′ GCAACGCGTTGTTGGCCATTTGTATGC 3′
1095 Forward - MluI Cut site	5′ CGGACGCGTTCTGGTAGTG 3′
893 Forward - MluI Cut site	5′ CGGACGCGTACTTGCTTGAG 3′
565 Forward - MluI Cut site	5′ CGGACGCGTTCTGGTAGTG 3′
294 Forward- MluI Cut site	5′ CCGACGCGTGTACAATAAAAGCG 3′

The table above provides detailed primers used for the truncation of the AMACR promoter. The same reverse primer was used do all the promoter cloning. The reverse primer, and full length forward primer were based on Chen *et al*. [1]

REFERENCES

1. Chen W, Wu W, Zhao J, Yu C, Liu W, Jiang A, Zhang J. Molecular cloning and preliminary analysis of the human alpha-methylacyl-CoA racemase promoter. Mol Biol Rep. 2009; 36:423–30. https://doi.org/10.1007/s11033-007-9196-x.
